# Alteration of ceruloplasmin in patients with malaria: a systematic review and meta-analysis of observational studies

**DOI:** 10.1186/s12936-024-05156-x

**Published:** 2024-11-21

**Authors:** Kwuntida Uthaisar Kotepui, Aongart Mahittikorn, Polrat Wilairatana, Frederick Ramirez Masangkay, Manas Kotepui

**Affiliations:** 1 Medical Technology Program, Faculty of Science, Nakhon Phanom University, Nakhon Phanom, 48000 Thailand; 2 Department of Protozoology, Faculty of Tropical Medicine, Mahidol University, Bangkok, 10400 Thailand; 3 Department of Clinical Tropical Medicine, Faculty of Tropical Medicine, Mahidol University, Bangkok, 10400 Thailand; 4 Department of Medical Technology, Faculty of Pharmacy, University of Santo Tomas, Manila, 1008 Philippines

**Keywords:** Malaria, Ceruloplasmin, Systematic review, Meta-analysis, Antioxidant, Oxidant

## Abstract

**Background:**

The evidences of oxidative stress-related *Plasmodium* infection may alter the ceruloplasmin levels were inconsistent. This systematic review and meta-analysis aimed to collate and synthesize literatures on malaria and ceruloplasmin concentrations.

**Methods:**

The systematic review has been registered with PROSPERO (CRD42023454859). Six electronic databases were systematically searched for investigated ceruloplasmin levels in malaria: ProQuest, EMBASE, MEDLINE, Ovid, PubMed, and Scopus, from their inception to August 2023. The quality of the included studies was assessed using the Joanna Briggs Institute (JBI) critical appraisal tools for cross-sectional, cohort, and case–control studies. Qualitative synthesis was undertaken to summarize findings from the included studies. For quantitative synthesis, a meta-analysis was performed using random-effects models.

**Results:**

A total of 411 articles were retrieved, and nine studies were included in the review. The majority of included studies found significantly increased ceruloplasmin levels in malaria patients compared to uninfected controls. The meta-analysis showed a significant increase of ceruloplasmin in patients with malaria as compared to uninfected controls (*P* < 0.01, Hedge’s g 1.18, 95% CI 0.90–1.47, *I*^*2*^ 59.19%, eight studies).

**Conclusion:**

The systematic review and meta-analysis consistently revealed a significant rise in ceruloplasmin levels among malaria patients. Further research is essential to understand the variations in ceruloplasmin levels between different *Plasmodium* species and the severity of malaria in patients.

**Supplementary Information:**

The online version contains supplementary material available at 10.1186/s12936-024-05156-x.

## Background

Malaria remains one of the most prevalent and deadly infectious diseases worldwide [1]. The disease results from infections by *Plasmodium* parasites and is primarily transmitted via bites from infected female *Anopheles* mosquitoes [2]. Predominantly afflicting tropical and sub-tropical regions, specific countries in sub-Saharan Africa and parts of Southeast Asia face the highest burden, with millions of individuals affected every year [3]. In 2021 alone, global estimates pinpointed approximately 247 million cases of malaria and a concerning 593,000 fatalities [3]. The complex pathophysiology of malaria involves myriad interactions at the cellular and systemic levels, necessitating a deep understanding for effective patient management and the development of innovative therapeutic strategies.

Ceruloplasmin, a copper-carrying ferroxidase enzyme, plays a critical role in iron homeostasis, inflammation, and antioxidant defense mechanisms [4]. Ceruloplasmin possesses antioxidant properties such as ferroxidase activity [5]. Furthermore, ceruloplasmin is an effective chain-breaking antioxidant for a variety of radicals such as peroxyl radical and superoxide radical [6]. In the contrary, the evidence suggested that ceruloplasmin may also exhibit potent pro-oxidant activity [7]. As an acute-phase protein, its concentration can oscillate in response to various pathological stimuli, marking it as a potential biomarker in a spectrum of diseases, including malaria [8]. Previous studies suggested that the oxidative stress instigated by malaria may catalyze an increase in ceruloplasmin levels [9, 10]. This raises intriguing questions about the potential diagnostic and prognostic role of ceruloplasmin in malaria-infected patients.

In light of the enduring burden of malaria and the promising implications of ceruloplasmin, there’s a palpable need to synthesize existing research for a more holistic understanding. This systematic review and meta-analysis endeavors to amalgamate the current literature, explore the nexus between malaria and ceruloplasmin concentrations, and discern patterns of clinical significance. By amalgamating this information, the study aimed to offer insights into ceruloplasmin’s role in malaria pathophysiology and assess its potential utility in clinical settings.

## Methods

### Review question

The systematic review question was formulated using the Population (P), Exposure (E), Comparator (C), and Outcome (O) framework [11]. In this context, ‘P’ refers to participants residing in malaria-endemic areas; ‘E’ pertains to individuals exposed to malaria; ‘C’ involves uninfected individuals from the same endemic areas; and ‘O’ is the level or presence of ceruloplasmin.

### Registration and guidelines

The systematic review has been registered with PROSPERO, an international database for the registration of systematic reviews (CRD42023454859). The reporting and presentation of the findings adhered to the Preferred Reporting Items for Systematic Reviews and Meta-Analyses (PRISMA) guidelines to ensure comprehensive and transparent reporting [12].

### Search strategy

Six electronic databases were systematically searched: ProQuest, EMBASE, MEDLINE, Ovid, PubMed, and Scopus, from their inception to August 2023. The search strategy comprised a combination of keywords and MeSH terms such as “ceruloplasmin” and “malaria” as “(Ceruloplasmin OR “Ceruloplasmin Oxidase” OR Ferroxidase OR “alpha(2)-Ceruloplasmin” OR “Ceruloplasmin Ferroxidase” OR “Ferroxidase I”) AND (malaria OR *Plasmodium* OR “*Plasmodium* Infection” OR “Remittent Fever” OR “Marsh Fever” OR Paludism)”. The search strategy slightly differed between databases (Table S1). A complementary search was executed on Google Scholar to capture articles outside these databases, and the reference lists of the retrieved articles were screened to identify additional eligible studies.

### Eligibility criteria

Studies were deemed eligible if they (i) investigated ceruloplasmin levels in malaria patients versus uninfected controls, (ii) were either cross-sectional, case-control, or cohort studies, (iii) clearly reported methods for diagnosing malaria and measuring ceruloplasmin. Conference abstracts, case reports, case series, reviews, in vivo or in vitro studies, and articles without ceruloplasmin data were excluded.

### Study selection and data extraction

Two independent reviewers screened titles and abstracts of the identified articles, and any discrepancies were resolved by consensus or by consulting a third reviewer. Subsequently, full-text articles were assessed for eligibility. From each eligible study, data on the following were extracted: author(s), publication year, study location, *Plasmodium* species, age range of participants, severity status, study design, diagnostic method for malaria, principle of the method used for ceruloplasmin measurement, and ceruloplasmin levels in both malaria and control groups.

### Quality assessment

The quality of the included studies was assessed using the Joanna Briggs Institute (JBI) critical appraisal tools for cross-sectional, cohort, and case-control studies [13]. For cross-sectional studies, the JBI focuses on the appropriateness of the sample frame, sampling methods, and size, the detailed description of study subjects and setting, the validity and reliability of condition measurement, and the provision of confidence intervals and sub-group analyses. Cohort studies are evaluated based on group comparability, exposure measurement consistency, detailed descriptions of subjects and setting, outcome measurement validity and reliability, follow-up time adequacy and completeness, and strategies to handle confounding factors. Case-control studies are appraised on the clarity of case and control definitions, accurate exposure measurement, case ascertainment methods, control selection methods, identification and management of confounding factors, and the appropriateness of statistical analyses. Each checklist question is typically answered with ‘Yes’, ‘No’, ‘Unclear’, or ‘Not applicable’, allowing for a systematic and consistent appraisal of study quality.

### Data synthesis and analysis

Qualitative synthesis was undertaken to summarize findings from the included studies. For quantitative synthesis, a meta-analysis was performed using random-effects models [14], with the Hedges’ g as the effect size to compare ceruloplasmin levels between malaria patients and uninfected controls. Heterogeneity was assessed using the *I*^*2*^ statistic, where values of 25%, 50%, and 75% indicated low, moderate, and high heterogeneity, respectively [15]. Meta-regression was employed to assess the potential sources of heterogeneity. Subgroup analyses were conducted based on factors like publication years, study designs, geographical locations, age ranges of participants, *Plasmodium* species, diagnostic methods for malaria, severity statuses, and the principle of methods used for ceruloplasmin measurement. Sensitivity analysis using the leave-one-out method was employed to assesses the robustness of results under varying conditions [16]. In this approach, one study is excluded from the analysis, and the analysis is rerun on the remaining data. By comparing the results of each rerun to the original findings, this method helps in recognizing influential studies, understanding result robustness, and detecting potential outliers. The funnel plot and Egger’s regression analysis were used to detect publication bias in a meta-analysis if the number of studies included was at least 10 studies aligning with the guidelines elsewhere [17]. All statistical analyses were carried out using Stata 18.0 software (Stata 18.0, College Station, TX: StataCorp LLC).

## Results

### Search results

**Fig. 1:**
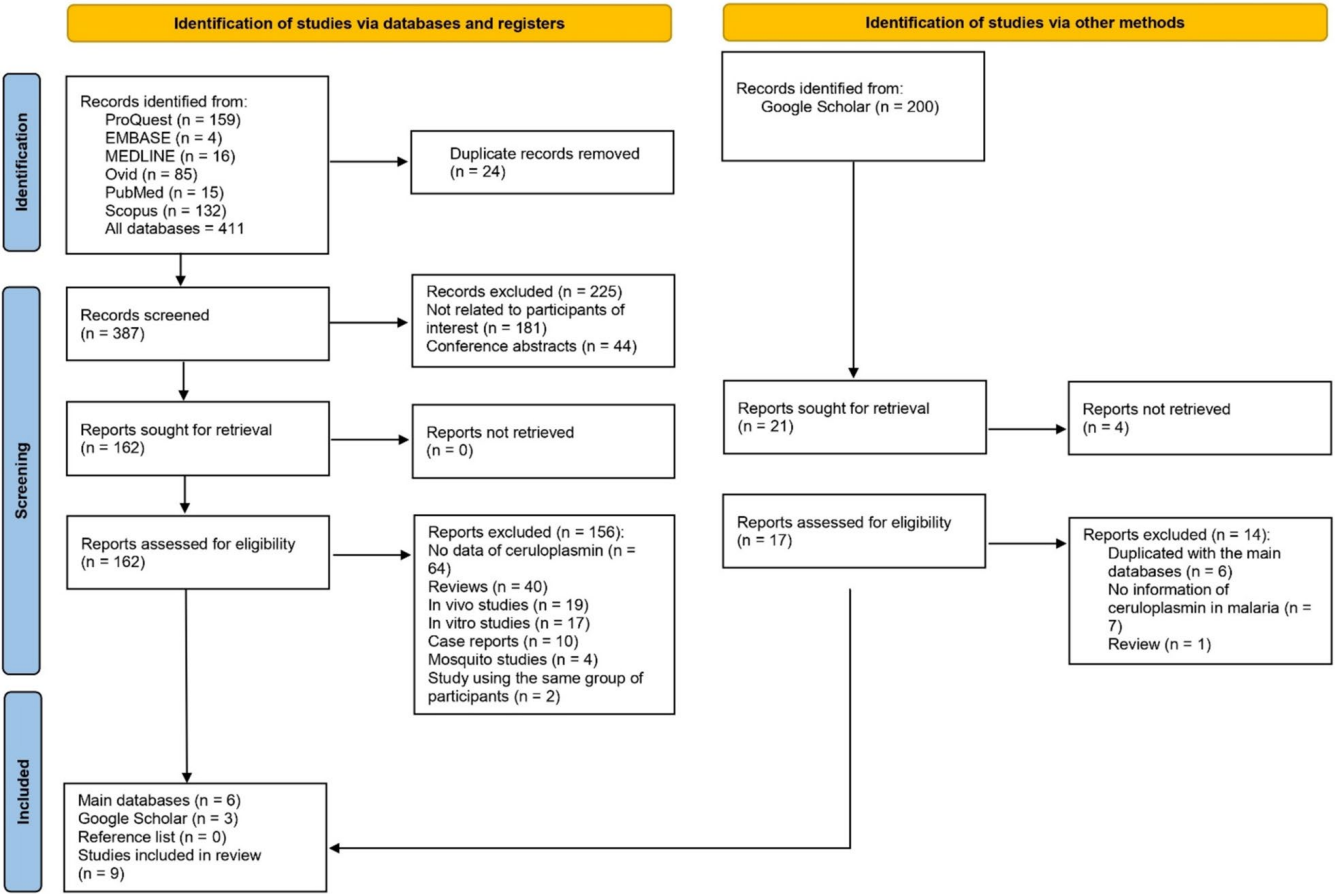
Study flow diagram showed the selection of relevant studies

Articles were initially identified from six databases: ProQuest (159 articles), EMBASE (4 articles), MEDLINE (16 articles), Ovid (85 articles), PubMed (15 articles), and Scopus (132 articles), resulting in a total of 411 articles. After the removal of 24 duplicate entries, 387 articles remained and were screened. Of these, 225 were excluded for various reasons, with 181 being irrelevant to the desired participants and 44 being conference abstracts. This left 162 articles for further assessment. However, 156 of them were excluded due to specific reasons, including the absence of ceruloplasmin data, and their nature as in vivo or in vitro studies, among others. Consequently, six articles from these databases met the eligibility criteria for the review [9, 18–22]. Additional searches in Google Scholar contributed three eligible articles [10, 23, 24], while reference lists yielded none. In total, nine studies were included in the review (Fig. 1) [9, 10, 18–24].

### Characteristics of included studies

Table 1 summarize the characteristics of the nine included studies. Categorized by publication year, three studies (33.33%) were dated prior to 2000, two studies (22.22%) between 2000 and 2009, and four studies (44.44%) spanned from 2010 to 2023. Examining Regarding the study design, five studies (55.56%) adopted a case-controlled approach, three studies (33.33%) were cross-sectional, and one study (11.11%) was cohort-based. Geographically, Asia dominated with seven studies (77.78%), specifically India, which contributed three studies (33.33%), followed by Turkey with two studies (22.22%). Nigeria and Brazil were each represented by a single study (11.11%). The presence of *Plasmodium* species was segmented into *Plasmodium falciparum* in five studies (55.56%), *Plasmodium vivax* in three studies (33.33%), and a combination in 1 study (11.11%). In terms of participant age groups, children were the focus in two studies (22.22%), adults in four studies (44.44%), and all age brackets were addressed in three studies (33.33%). The distribution based on severity indicated three studies (33.33%) on non-severe malaria, two studies (22.22%) on severe malaria, one study (11.11%) considering both types, and three studies (33.33%) remaining unspecified. Diagnostic modalities predominantly used microscopy in seven studies (77.78%), with an integrative approach, combining microscopy with either PCR or RDT, in one study each (11.11% respectively). Methodological principles for ceruloplasmin measurement included oxidase activity in five studies (55.56%), ceruloplasmin activity in two studies (22.22%), colorimetric assay, and immunodiffusion, both in one study (11.11% respectively). Detailed characteristics of each study are presented in Table 2.

**Table 1:** Characteristics of studies

Characteristics	*n*. (9 studies)	%
Publication year		
Before 2000	3	33.33
2000–2009	2	22.22
2010–2023	4	44.44
Study designs		
Case-control studies	5	55.56
Cross-sectional studies	3	33.33
Cohort study	1	11.11
Study areas		
Asia	7	77.78
India	3	33.33
Turkey	2	22.22
Thailand	1	11.11
Yemen	1	11.11
Africa (Nigeria)	1	11.11
South America (Brazil)	1	11.11
Plasmodium spp.		
*P. falciparum*	5	55.56
*P. vivax*	3	33.33
*P. falciparum*,* P. vivax*	1	11.11
Participants		
Children	2	22.22
Adults	4	44.44
All age groups	3	33.33
Severity status		
Non-severe malaria	3	33.33
Severe malaria	2	77.78
Severe and non-severe malaria	1	11.11
Not specified	3	33.33
Methods for malaria detection		
Microscopy	7	77.78
Microscopy/PCR	1	11.11
Microscopy/RDT	1	11.11
Principle of methods for ceruloplasmin measurement		
Oxidase activity	5	55.56
Ceruloplasmin activity	2	22.22
Colorimetric assay	1	11.11
Immunodiffusion	1	11.11

*RDT* rapid diagnostic test, *PCR* polymerase chain reaction

**Table 2:** Characteristics and results of individual study included in the review

No.	Authors	Study location	*Plasmodium* spp.	Age range	Severity status	Qualitative synthesis of ceruloplasmin levels
1	Das et al. (1996) [9]	India	*P. falciparum*	Children	Severe and non-severe	(1) Ceruloplasmin levels were significantly increased in malaria (severe or non-severe) patients as compared to uninfected controls. (2) Ceruloplasmin levels were significantly increased in severe malaria as compared to non-severe malaria
2	Erel et al. (1997) [10]	Turkey	*P. vivax*	Adults	Severe	Ceruloplasmin levels were significantly increased in malaria patients as compared to uninfected controls
3	Ezzi et al. (2017) [23]	Yemen	*P. falciparum*	Adults	Non-severe	Ceruloplasmin levels were significantly increased in malaria patients as compared to uninfected controls
4	Fabbri et al. (2013) [18]	Brazil	*P. vivax*	All age ranges	Non-severe	Ceruloplasmin levels were significantly increased in malaria patients as compared to uninfected controls
5	Farombi et al. (2003) [19]	Nigeria	*P. falciparum*	Adults	Non-severe	Ceruloplasmin levels were significantly increased in malaria patients as compared to uninfected controls
6	Kamble et al. (2011) [24]	India	*P. falciparum/P. vivax*	Adults	Not specified	No difference in ceruloplasmin between malaria and uninfected controls
7	Narsaria et al. (2012) [20]	India	*P. falciparum*	Children	Severe	Ceruloplasmin levels were significantly decreased in malaria patients as compared to uninfected controls
8	Seyrek et al. (2005) [21]	Turkey	*P. vivax*	All age ranges	Not specified	Ceruloplasmin levels were significantly increased in malaria patients as compared to uninfected controls
9	Thurnham et al. (1990) [22]	Thailand	*P. falciparum*	All age ranges	Not specified	Ceruloplasmin levels were significantly increased in malaria patients as compared to uninfected controls

### Quality of included studies

In the case of the case-control studies [10, 20–22, 24], all four—Erel et al. [10]; Kamble et al. [24]; Narsaria et al. [20]; Seyrek et al. [21] and Thurnham et al. [22]—satisfied the majority of the outlined criteria. However, all studies were deficient in reporting the exposure period of interest. For the cross-sectional studies [9, 18, 23], all four—Das et al. [9]; Ezzi et al. [23] and Fabbri et al. [18]—met most of the criteria. Regarding the cohort study, Farombi et al. [19] was ambiguous concerning strategies to address confounding factors, the duration of follow-up, and the reasons for loss to follow-up (Table S2).

### Qualitative synthesis

In India, Das et al. studied children infected with *P. falciparum*, both with severe and non-severe malaria, and found significantly increased ceruloplasmin levels in malaria patients compared to uninfected controls [9]. Specifically, there was a more significant increase in ceruloplasmin levels in cases of severe malaria than non-severe cases. Meanwhile, the study by Erel et al. in Turkey on adults infected with *P. vivax* having severe malaria also reported increased ceruloplasmin levels [10]. Similarly, in Yemen, Ezzi et al. found that adults with non-severe malaria caused by *P. falciparum* showed increased ceruloplasmin levels [23]. In Brazil, Fabbri et al. examined *P. vivax* infection across all age ranges with non-severe malaria and found the same increase [18]. The study by Farombi et al. in Nigeria on adults with non-severe *P. falciparum* malaria echoed these findings [19]. Seyrek et al.. in Turkey and Thurnham et al. in Thailand, studying *P. vivax* and *P. falciparum* infections respectively across all age ranges with unspecified clinical severity, also reported similar increases in ceruloplasmin levels [21, 22].

Conversely, in India, Narsaria et al. observed a decrease in ceruloplasmin levels in children with severe malaria caused by *P. falciparum* [20]. Finally, the study by Kamble et al., also in India, involving adults with either *P. falciparum* or *P. vivax* infection and unspecified clinical severity, found no difference in ceruloplasmin levels between the malaria patients and the controls [24] (Table 2).

### Meta-analysis

**Fig. 2:**
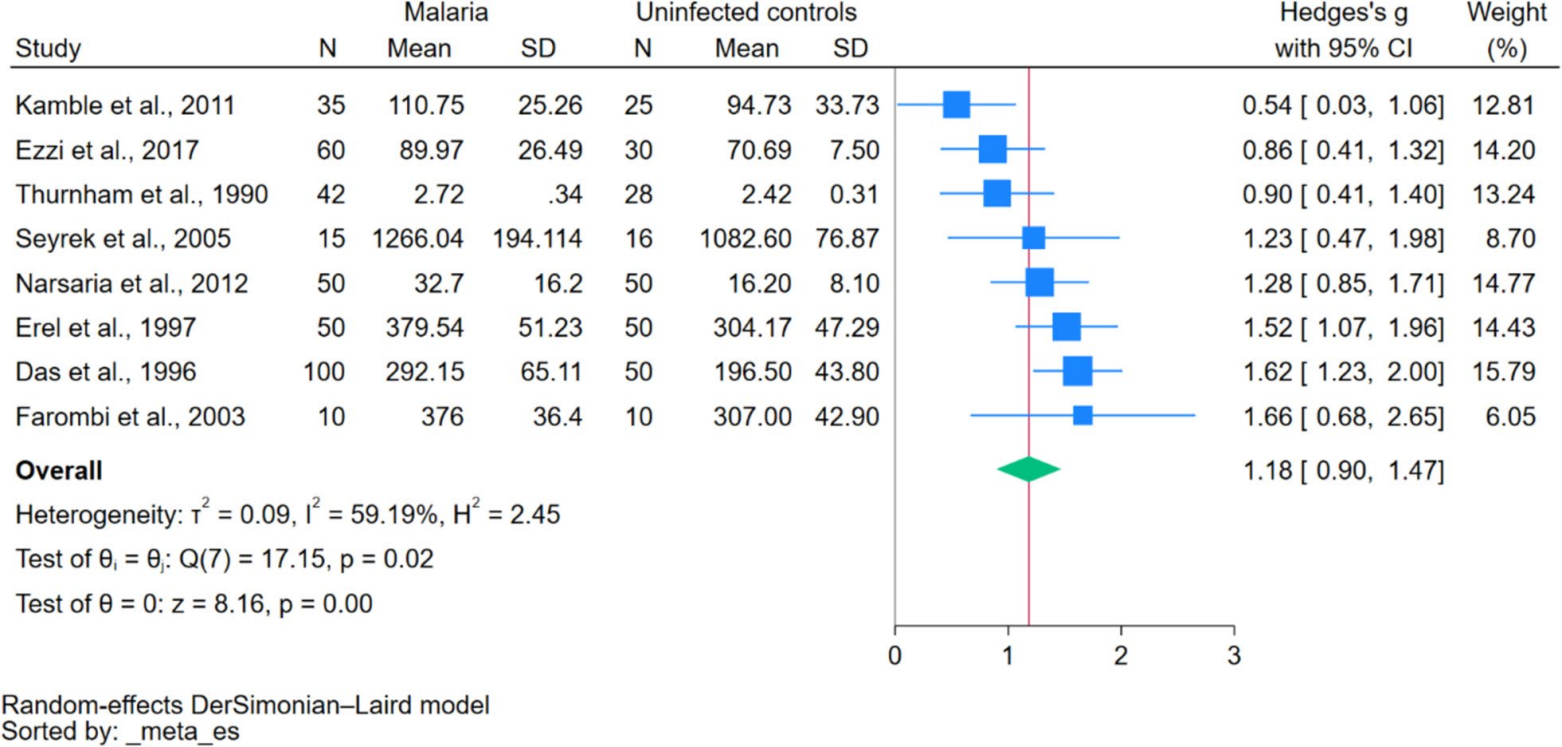
The forest plot of meta-analysis showed a significant increase of ceruloplasmin in patients with malaria as compared to uninfected controls. Explanations of symbols: blue square, effect estimates; blue vertical line, confidence interval; green diamond, pooled effect estimate; gray horizontal line, no effect line; red horizontal line, pooled effect line; N, number pf participants in each group; CI, confidence interval; SD, standard deviation

The meta-analysis was performed using the data from eight studies [9, 10, 19–24]. One study did not specify the quantitative data of ceruloplasmin [18]. The meta-analysis showed a significant increase of ceruloplasmin in patients with malaria as compared to uninfected controls (*P* < 0.01, Hedge’s g 1.18, 95% CI 0.90–1.47, *I*^2^ 59.19%, eight studies, Fig. 2). There was a high heterogeneity of the effect estimate in the results of the meta-analysis (*I*^2^ 59.19%). The meta-regression analysis was subsequently performed, and the results showed the severity status of patients affected the pooled effect estimate (*P* = 0.036, Table 3).

**Table 3:** Meta-regression results

Meta-analysis of ceruloplasmin	Covariates	*P* value	tau^2^	I^2^ (%)	*R*-squared (%)	Number of studies
Malaria patients vs. uninfected individuals	Publication years	0.229	0.069	52.83	26.54	8
	Study designs	0.654	0.126	67.49	0.00	8
	Continents	0.404	0.099	63.17	0.00	8
	Age ranges	0.462	0.095	58.03	0.00	8
	*Plasmodium* species	0.129	0.056	46.00	40.06	8
	Diagnostic method for malaria	0.802	0.126	64.72	0.00	8
	Severity status	0.036	0.019	19.70	79.29	8
	Principle of methods for ceruloplasmin measurement	0.782	0.162	68.74	0.00	8

The subgroup analysis showed a comprehensive subgroup analysis, shedding light on variations in ceruloplasmin levels between malaria-infected individuals and uninfected controls, categorized by several factors. Notably, during different publication years, there was an elevation in ceruloplasmin levels in malaria patients in all year ranges including before 2000 (*P* < 0.01, Hedges’ g: 1.37, 95% CI: 0.95–1.78), 2000–2009 (*P* < 0.01, Hedges’ g: 1.39, 95% CI: 0.79–1.98), and 2010–2023 (*P* < 0.01, Hedges’ g: 0.91, 95% CI: 0.50–1.33). With different study designs, there was an elevation in ceruloplasmin levels in malaria patients in cross-sectional studies (*P* < 0.01, Hedges’ g: 1.25, 95% CI: 0.51–1.99), and case-control studies (*P* < 0.01, Hedges’ g: 1.10, 95% CI: 0.75–1.45). Geographically, there was an elevation in ceruloplasmin levels in malaria patients in studies from Asia (*P* < 0.01, Hedges’ g: 1.15, 95% CI: 0.85–1.45). Age-wise disparities were evident, especially in children (*P* < 0.01, Hedges’ g: 1.46, 95% CI: 1.13–1.79). For different *Plasmodium* species, there was an elevation in ceruloplasmin levels in studies that enrolled patients with *P. falciparum* (*P* < 0.01, Hedges’ g: 1.23, 95% CI: 0.90–1.56), and *P. vivax* (*P* < 0.01, Hedges’ g: 1.44, 95% CI: 1.06–1.82). Based on malaria severity, there was an elevation in ceruloplasmin levels in studies enrolled patients with severe malaria (*P* < 0.01, Hedges’ g: 1.39, 95% CI: 1.09–1.70), and non-severe malaria (*P* < 0.01, Hedges’ g: 1.14, 95% CI: 0.40–1.88). Ceruloplasmin levels also varied with diagnostic methods, and the specific approach method used for ceruloplasmin measurement (Table 4).

**Table 4:** Subgroup analyses of ceruloplasmin levels between malaria cases and uninfected controls

Subgroup analyses	*P* value	Hedges’ g (95% CI)	I^2^	Number of studies
Publication years				
Before 2000	< 0.01	1.37 (0.95–1.78)	62.58	3
2000–2009	< 0.01	1.39 (0.79–1.98)	0.00	2
2010–2023	< 0.01	0.91 (0.50–1.33)	58.15	3
Study design				
Cross-sectional study	< 0.01	1.25 (0.51–1.99)	83.74	2
Case-control study	< 0.01	1.10 (0.75–1.45)	56.67	5
Cohort study	N/A	1.66 (0.68–2.65)	N/A	1
Continent				
Asia	< 0.01	1.15 (0.85–1.45)	63.17	7
Africa	N/A	1.66 (0.68–2.65)	N/A	1
Age ranges				
Children	< 0.01	1.46 (1.13–1.79)	24.25	2
Adults	< 0.01	1.09 (0.59–1.60)	70.29	4
All age groups	< 0.01	1.00 (0.59–1.41)	0.00	2
*Plasmodium* species				
*P. falciparum*	< 0.01	1.23 (0.90–1.56)	54.70	5
*P. vivax*	< 0.01	1.44 (1.06–1.82)	0.00	2
*P. falciparum*,* P. vivax*	N/A	0.54 (0.03–1.06)	N/A	1
Severity status				
Severe malaria	< 0.01	1.39 (1.09–1.70)	0.00	2
Non-severe malaria	< 0.01	1.14 (0.40–1.88)	51.83	2
Severe and non-severe malaria	N/A	1.62 (1.23–2.00)	N/A	1
Not specified	< 0.01	0.83 (0.48–1.18)	14.04	3
Diagnostic method for malaria				
Microscopy	< 0.01	1.17 (0.83–1.50)	64.72	7
Microscopy/RDT	N/A	1.28 (0.85–1.71)	N/A	1
Principle of methods for ceruloplasmin measurement				
Oxidase activity	< 0.01	1.31 (0.78–1.84)	75.29	4
Ceruloplasmin activity	< 0.01	0.96 (0.57–1.35)	0.00	2
Colorimetric assay	N/A	1.28 (0.85–7.71)	N/A	1

*CI* confidence interval, *N/A* not assessed, *RDT* rapid diagnostic test

### Sensitivity analysis

**Fig. 3:**
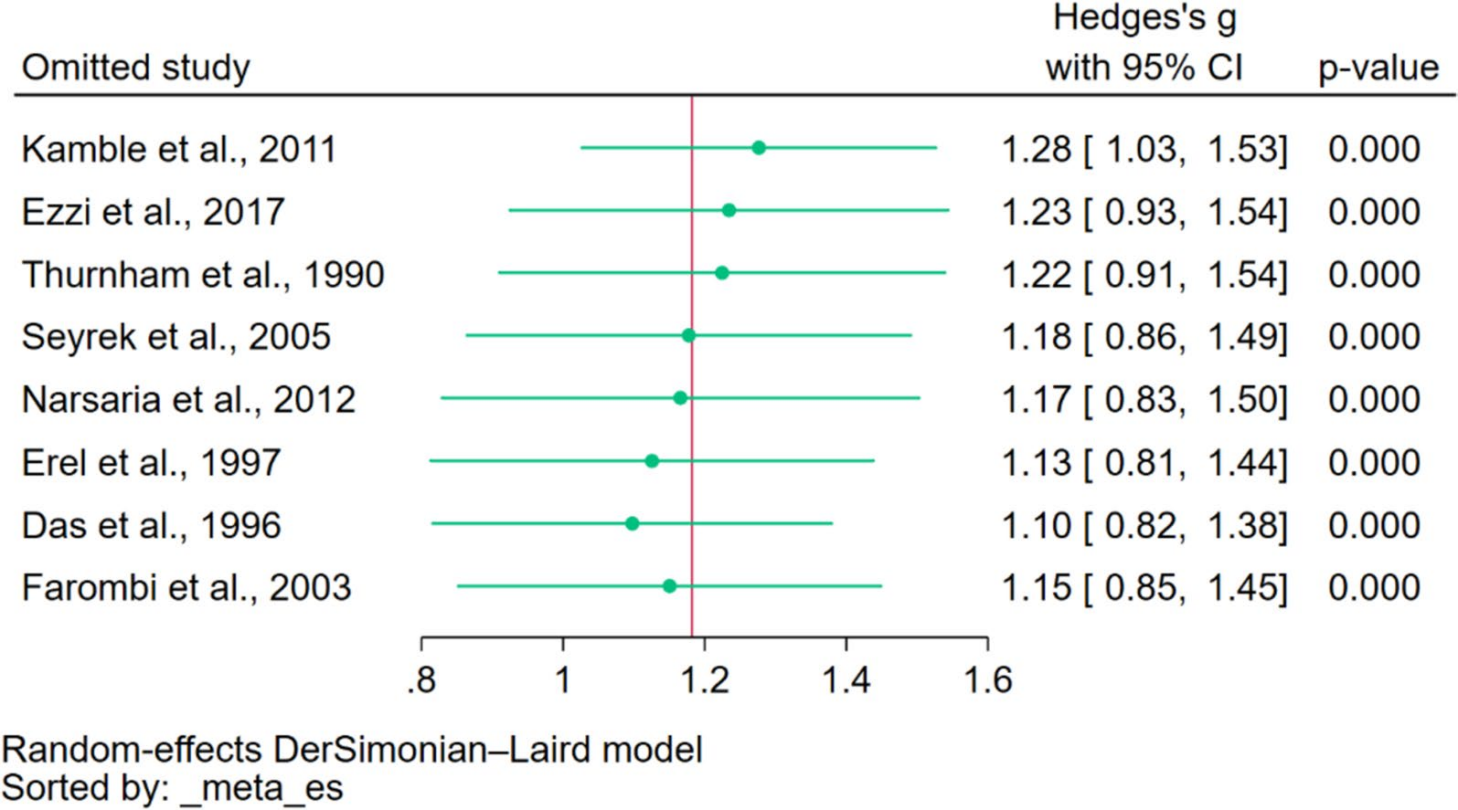
The leave-one-out meta-analysis demonstrated the stability and reliability of the meta-analysis as when each study was systematically excluded from the meta-analysis and the analysis re-conducted, the consistent observation was that ceruloplasmin levels remained elevated in individuals with malaria compared to those without the infection across all iterations. Explanations of symbols: green dots, pooled effect estimate; green vertical line, confidence interval; CI, confidence interval

The leave-one-out sensitivity analysis, a technique in meta-analysis, involves omitting one study at a time and recalculating the combined results. In the analysis, when each study was systematically excluded from the meta-analysis and the analysis re-conducted, the consistent observation was that ceruloplasmin levels remained elevated in individuals with malaria compared to those without the infection across all iterations (Fig. 3). Such consistent findings across multiple recalculations underscore the stability and reliability of the meta-analysis, suggesting that the conclusion drawn was robust and not overly dependent on any single study included in the analysis.

### Publication bias

The assessment of publication bias was not undertaken in this study. This decision was based on the recommendations which suggest that when the number of studies included in a meta-analysis is fewer than 10, the power to detect and interpret funnel plot asymmetry becomes significantly low, leading to potential misinterpretations. In the meta-analysis, as eight studies were incorporated, aligning with the guidelines elsewhere [17].

## Discussion

Ceruloplasmin is a copper-carrying ferroxidase enzyme that plays a pivotal role in iron homeostasis and antioxidant defense mechanisms [4]. In the current meta-analysis, an evident increase in ceruloplasmin levels was observed in patients diagnosed with malaria as compared to the uninfected controls. This was consistent with the known role of ceruloplasmin as an acute phase reactant, whose levels may rise in response to inflammation and infection [25]. However, the significant heterogeneity observed in this meta-analysis (*I*^*2*^: 59.19%) underscores the need for cautious interpretation. Heterogeneity in meta-analyses can arise from a variety of sources including methodological differences across studies, diverse patient populations, differences in *Plasmodium* species and the stages of infection, among others. Interestingly, the meta-regression analysis further identified that the severity status of patients played a role in influencing ceruloplasmin levels. This suggests that as the malaria infection progresses or becomes more severe, the body’s inflammatory response, and consequently ceruloplasmin levels, may vary. It had been postulated that ceruloplasmin acts as a defense mechanism to combat the oxidative stress [26]. In the contrary, another study suggested that ceruloplasmin acts as an antioxidative biomarker which controls oxidative stress [27].

Ceruloplasmin is widely recognized as an acute-phase protein with a multifaceted role in iron homeostasis, antioxidant defense mechanisms, and inflammation [8]. This subgroup analysis offers a more granular understanding of how ceruloplasmin levels vary in patients with malaria compared to uninfected controls, with regard to several factors. The elevation of ceruloplasmin levels in malaria patients across different publication years underscores the consistency of the association between malaria and increased ceruloplasmin. This might be indicative of the fact that despite advancements in diagnostic and therapeutic strategies over the years, the inflammatory response of the body to malaria remains relatively consistent [28]. Differences in study design, such as cross-sectional and case-control studies, both show elevated levels of ceruloplasmin in malaria-affected individuals. This is pertinent as it illustrates that regardless of the study design, the association was consistently observed, thus fortifying the validity of the findings.

Regionally, elevated ceruloplasmin levels observed in Asian studies could be influenced by non-infectious diseases prevalent in the area, such as autism spectrum disorder [29], type II diabetes mellitus [30], and Alzheimer’s disease [31]. Similarly, in African studies, elevated ceruloplasmin levels were observed in conditions like cardiomyopathy [32], heart failure [33], and venous thromboembolism [34]. Notably, only one study from Africa reported an elevation of ceruloplasmin levels in malaria patients who did not receive anti-malarial therapy [19]. Interestingly, patients with malaria who were treated with chloroquine demonstrated a more significant increase in ceruloplasmin levels compared to those who did not receive any antimalarial therapy [19]. This suggests that ceruloplasmin might act as an oxidative stress marker. Since only one study from Africa was included in the meta-analysis, further research is needed to elucidate ceruloplasmin levels in malaria patients from the African region.

The marked difference in ceruloplasmin levels among children suffering from malaria is concerning. Given that children are particularly susceptible to severe malaria [35, 36], the elevated ceruloplasmin could potentially be explored as a marker for disease severity or progression in pediatric populations. A previous study indicated that serum ceruloplasmin levels are naturally minimal during early infancy up to 6 months of age, peak beyond adult levels in early childhood, and then stabilize within the adult range [37]. Consequently, the pronounced variations in ceruloplasmin levels in the subgroup analysis of age groups might be influenced by the baseline levels of ceruloplasmin in different age groups. In the subgroup analysis of *Plasmodium* species, no distinction was observed in ceruloplasmin levels between *P. falciparum* and *P. vivax*. This suggests that the elevated ceruloplasmin levels in malaria patients are independent of the specific *Plasmodium* species. Although *P. falciparum* is known to trigger a strong inflammatory response leading to severe malaria pathophysiology [28], recent evidence suggests that *P. vivax* may elicit even greater inflammation [38–40]. Further studies are warranted to elucidate the differences in ceruloplasmin levels between these two *Plasmodium* species.

Significant variations in ceruloplasmin levels were observed between patients with malaria and uninfected individuals, with differences based on the severity of malaria. Erel et al., who examined ceruloplasmin in patients with severe vivax malaria, suggested that an increase in ceruloplasmin might indicate its oxidant properties in vivax malaria cases [10]. In contrast, a study by Narsaria et al., which included patients with severe falciparum malaria, showed a significant decrease in ceruloplasmin levels among those patients [20]. These findings hint at potentially different roles of ceruloplasmin during *P. falciparum* and *P. vivax* infections. It is possible that ceruloplasmin acts as an oxidant in *P. vivax* infections and as an antioxidant in *P. falciparum* infections [10, 20].

The systematic review and meta-analysis presented several limitations that could potentially influence its outcomes. Firstly, many studies lacked comprehensive reporting on the exposure period, which could lead to ambiguity in ceruloplasmin assessment in relation to the disease progression. The meta-analysis was based on a limited number of studies, eight in total, making it challenging to assess publication bias or conduct certain subgroup analyses. Moreover, despite geographical diversity in the studies considered, the overall patient pool might not represent the global malaria population adequately. Lastly, variations in methodologies across studies, such as sample collection and ceruloplasmin measurement techniques, might introduce heterogeneity, which could affect the pooled results’ accuracy.

Given the observed association between ceruloplasmin levels and malaria infection severity, there are noteworthy implications for clinical practice. Monitoring ceruloplasmin levels could serve as a valuable biomarker to gauge disease severity and progression in patients with malaria. Early detection of alterations in ceruloplasmin levels might facilitate timely medical interventions, potentially improving patient outcomes. Moreover, understanding this association could pave the way for therapeutic research focusing on modulating ceruloplasmin levels to potentially mitigate the severity of the infection. However, clinicians should approach the use of ceruloplasmin as a biomarker cautiously, considering the limitations of the current evidence and ensuring they account for other confounding factors in the clinical setting.

## Conclusion

The systematic review and meta-analysis consistently revealed a significant rise in ceruloplasmin levels among malaria patients. The study also demonstrated that the increase in ceruloplasmin levels corresponded with the severity of the infection. Monitoring ceruloplasmin might provide early indications of disease progression, allowing for timely medical interventions that could improve patient outcomes. Using ceruloplasmin as a biomarker could be invaluable in assessing disease severity in malaria patients. Further research is essential to understand the variations in ceruloplasmin levels between different *Plasmodium* species and the severity of malaria in patients.

## Supplementary Information

Supplementary Material 1.

Supplementary Material 2.

Supplementary Material 3.

## Data Availability

All data relating to the present study are available in this manuscript and supplementary files.
