# Supplementary material for: Alteration of ceruloplasmin in patients with malaria: a systematic review and meta-analysis of observational studies
Source: Malar J. 2024 Nov 21;23:353. doi: 10.1186/s12936-024-05156-x (PMC11580669; doi:10.1186/s12936-024-05156-x)
Supplement: Supplementary file 1 — Supplementary Material 1. [file 12936_2024_5156_MOESM1_ESM.docx]

**General keywords**

(Ceruloplasmin OR “Ceruloplasmin Oxidase” OR Ferroxidase OR “alpha(2)-Ceruloplasmin” OR “Ceruloplasmin Ferroxidase” OR “Ferroxidase I”) AND (malaria OR plasmodium OR “Plasmodium Infection“ OR “Remittent Fever“ OR “Marsh Fever“ OR Paludism)

PubMed 21 July 2023

| No. | Key concept | Search terms | Results |
| --- | --- | --- | --- |
| 1. | Ceruloplasmin | Ceruloplasmin[Text Word] OR Ceruloplasmin[MeSH Terms] OR Ferroxidase[Text Word] OR “Ceruloplasmin Oxidase”[Text Word] OR Ferroxidase I[Text Word] OR “alpha(2)-Ceruloplasmin”[Text Word] | 9,692 |
| 2. | Malaria | malaria[Text Word] OR plasmodium[Text Word] OR malaria[MeSH Terms] OR “Remittent Fever“[Text Word] OR “Marsh Fever“[Text Word] OR Paludism[Text Word] | 118,784 |
| 3. | 1 AND 2 | (Ceruloplasmin[Text Word] OR Ceruloplasmin[MeSH Terms] OR Ferroxidase[Text Word] OR “Ceruloplasmin Oxidase”[Text Word] OR Ferroxidase I[Text Word] OR “alpha(2)-Ceruloplasmin”[Text Word]) AND (malaria[Text Word] OR plasmodium[Text Word] OR malaria[MeSH Terms] OR “Remittent Fever“[Text Word] OR “Marsh Fever“[Text Word] OR Paludism[Text Word]) | 15 |

Embase 24 July 2023

| No. | Key concept | Search terms | Results |
| --- | --- | --- | --- |
| 1. | Ceruloplasmin | Ceruloplasmin:ti,ab,kw,de OR Ceruloplasmin/exp OR Ferroxidase:ti,ab,kw,de OR “Ceruloplasmin Oxidase”:ti,ab,kw,de OR Ferroxidase I:ti,ab,kw,de OR “alpha(2)-Ceruloplasmin”:ti,ab,kw,de | 1274 |
| 2. | Malaria | malaria:ti,ab,kw,de OR plasmodium:ti,ab,kw,de OR ‘Remittent Fever’:ti,ab,kw,de OR ‘Marsh Fever’:ti,ab,kw,de OR Paludism:ti,ab,kw,de OR malaria/exp | 156,879 |
| 3. | 1 AND 2 | (Ceruloplasmin:ti,ab,kw,de OR Ceruloplasmin/exp OR Ferroxidase:ti,ab,kw,de OR “Ceruloplasmin Oxidase”:ti,ab,kw,de OR Ferroxidase I:ti,ab,kw,de OR “alpha(2)-Ceruloplasmin”:ti,ab,kw,de) AND (malaria:ti,ab,kw,de OR plasmodium:ti,ab,kw,de OR ‘Remittent Fever’:ti,ab,kw,de OR ‘Marsh Fever’:ti,ab,kw,de OR Paludism:ti,ab,kw,de OR malaria/exp) | 4 |

Scopus 24 July 2023

| No. | Key concept | Search terms | Results |
| --- | --- | --- | --- |
| 1. | Ceruloplasmin | TITLE-ABS-KEY (urate OR uric OR “uric acid” OR “2,6,8-trihydroxypurine” OR trioxopurine) | 82376 |
| 2. | Malaria | TITLE-ABS-KEY ( ( malaria OR plasmodium OR "plasmodium infection" OR "remittent fever" OR "marsh fever" OR paludism ) ) | 157,694 |
| 3. | 1 AND 2 | ( TITLE-ABS-KEY ( urate OR uric OR "uric acid" OR "2,6,8-trihydroxypurine" OR trioxopurine ) ) AND ( TITLE-ABS-KEY ( ( malaria OR plasmodium OR "Plasmodium Infection" OR "Remittent Fever" OR "Marsh Fever" OR paludism ) ) ) | 132 |

MEDLINE 24 July 2023

| No. | Key concept | Search terms | Results |
| --- | --- | --- | --- |
| 1. | Ceruloplasmin AND Malaria | (Ceruloplasmin OR “Ceruloplasmin Oxidase” OR Ferroxidase OR “alpha(2)-Ceruloplasmin” OR “Ceruloplasmin Ferroxidase” OR “Ferroxidase I”) AND (malaria OR plasmodium OR “Plasmodium Infection“ OR “Remittent Fever“ OR “Marsh Fever“ OR Paludism) | 16 |

Ovid 24 July 2023

| No. | Key concept | Search terms | Results |
| --- | --- | --- | --- |
| 1. | Ceruloplasmin AND Malaria | (Ceruloplasmin OR “Ceruloplasmin Oxidase” OR Ferroxidase OR “alpha(2)-Ceruloplasmin” OR “Ceruloplasmin Ferroxidase” OR “Ferroxidase I”) AND (malaria OR plasmodium OR “Plasmodium Infection“ OR “Remittent Fever“ OR “Marsh Fever“ OR Paludism)  Filters: (ovid full text available and articles with abstracts and original articles) | 85 |

ProQuest 24 July 2023

| No. | Key concept | Search terms | Results |
| --- | --- | --- | --- |
| 1. | Ceruloplasmin AND Malaria | (Ceruloplasmin OR “Ceruloplasmin Oxidase” OR Ferroxidase OR “alpha(2)-Ceruloplasmin” OR “Ceruloplasmin Ferroxidase” OR “Ferroxidase I”) AND (malaria OR plasmodium OR “Plasmodium Infection“ OR “Remittent Fever“ OR “Marsh Fever“ OR Paludism) | 159 |

**Google Scholar**

**15 August 2023**

(Ceruloplasmin OR “Ceruloplasmin Oxidase” OR Ferroxidase OR “alpha(2)-Ceruloplasmin” OR “Ceruloplasmin Ferroxidase” OR “Ferroxidase I”) AND (malaria OR plasmodium OR “Plasmodium Infection“ OR “Remittent Fever“ OR “Marsh Fever“ OR Paludism)
